# *Origanum majorana* Essential Oil Lacks Mutagenic Activity in the* Salmonella*/Microsome and Micronucleus Assays

**DOI:** 10.1155/2016/3694901

**Published:** 2016-11-07

**Authors:** Andrea dos Santos Dantas, Luiz Carlos Klein-Júnior, Miriana S. Machado, Temenouga N. Guecheva, Luciana D. dos Santos, Régis A. Zanette, Fernanda B. de Mello, João Antonio Pêgas Henriques, João Roberto Braga de Mello

**Affiliations:** ^1^Programa de Pós-Graduação em Ciências Veterinárias, UFRGS, Porto Alegre, RS, Brazil; ^2^Laboratório de Produtos Naturais/Fitoquímica-Farmacologia e Toxicologia Veterinária, Departamento de Farmacologia, UFRGS, Porto Alegre, RS, Brazil; ^3^Laboratório de Farmacognosia e Controle de Qualidade de Fitomedicamentos, Faculdade de Farmácia, UFRGS, Porto Alegre, RS, Brazil; ^4^Laboratório de Reparação de DNA em Eucariotos, Departamento de Biofísica/Centro de Biotecnologia, UFRGS, Porto Alegre, RS, Brazil; ^5^Programa de Pós-Graduação em Farmacologia e Terapêutica, UFRGS, Porto Alegre, RS, Brazil

## Abstract

The present study aimed to investigate the* in vitro* mutagenic activity of* Origanum majorana* essential oil. The most abundant compounds identified by GC-MS were *γ*-terpinene (25.73%), *α*-terpinene (17.35%), terpinen-4-ol (17.24%), and sabinene (10.8%). Mutagenicity was evaluated by the* Salmonella*/microsome test using the preincubation procedure on TA98, TA97a, TA100, TA102, and TA1535* Salmonella typhimurium* strains, in the absence or in the presence of metabolic activation. Cytotoxicity was detected at concentrations higher than 0.04 *μ*L/plate in the absence of S9 mix and higher than 0.08 *μ*L/plate in the presence of S9 mix and no gene mutation increase was observed. For the* in vitro* mammalian cell micronucleus test, V79 Chinese hamster lung fibroblasts were used. Cytotoxicity was only observed at concentrations higher than or equal to 0.05 *μ*g/mL. Moreover, when tested in noncytotoxic concentrations,* O. majorana* essential oil was not able to induce chromosome mutation. The results from this study therefore suggest that* O. majorana* essential oil is not mutagenic at the concentrations tested in the* Salmonella*/microsome and micronucleus assays.

## 1. Introduction

The* Origanum* genus belongs to the Lamiaceae family and includes species with interesting pharmacological effects [1].* Origanum majorana *L. or* Majorana hortensis *Moench is an aromatic plant rich in essential oils and commercially grown in southern Europe and in the Mediterranean region [2, 3]. It is popularly known as marjoram and has been used in the form of herbal infusions in folk medicine for asthma, cold, coughs, cramps, depression, dizziness, gastrointestinal disorders, hay fever, headache, toothache, and sinus congestion and as a diuretic and to promote menstruation [2, 3].

*O. majorana* crude extract, dichloromethane, ethyl acetate, aqueous fractions, and* O. majorana* essential oil have shown significant results in inhibiting the growth of bacteria and fungi and the synthesis of microbial metabolites [1, 4, 5]. Because of its antioxidant effects [6–8],* O. majorana* essential oil or extract can be used in the prevention of central nervous system disorders [2, 9].* O. majorana* essential oil was also able to partially prevent the ethanol-induced decline in sperm quality, testosterone levels, and the weight of reproductive organs in male rats [2]. Previous studies have reported the potential use of* O. majorana* ethanolic extract as anticancer agent [10, 11], whereas the tea extract has been shown to have immunostimulant, antigenotoxic and antimutagenic properties [3, 12]. These activities are attributed to the chemical composition, which is characterized as rich in flavonoids and terpenoids [13].

In spite of the growing interest in using essential oils and other extracts in the treatment of diseases, it is necessary to perform toxicological studies to ensure that the chemical compounds found in the plant under study exert no adverse effects that would impair its use for therapeutic purposes. Genetic toxicology data are employed as a surrogate for long-term carcinogenicity data during early drug development and must be conducted when there is an indication of continuous use or prolonged therapeutic treatment. The genotoxicity battery includes gene and chromosome mutation detection tests for products for human use [14, 15].

Therefore, this study was performed to investigate the mutagenic activity of* O. majorana* essential oil using five strains of bacteria in the* Salmonella*/microsome test, with or without metabolic activation, and in the* in vitro* mammalian cell micronucleus test using Chinese hamster lung fibroblasts (V79 cells). Essential oil chemical characterization was also performed to identify the major components of the oil.

## 2. Material and Methods

### 2.1. Chemicals

Diethyl ether was acquired from Tedia Company, Inc. (Fairfield, OH, USA). Aflatoxin B1 (CAS 1162-65-8), 2-aminofluorene (CAS 153-78-6), 4-nitroquinoline-oxide (CAS 56-57-5), sodium azide (CAS 26628-22-8), colchicine (CAS 64-86-8), etoposide (CAS 33419-42-0), cytochalasin B (14930-96-2), dimethyl sulfoxide (DMSO) (CAS 67-68-5), glucose-6-phosphate (CAS 56-73-5), and NADP (CAS 698-999-85-8) were obtained from Sigma Chemical (St. Louis, USA). Dulbecco's Modified Eagle's Medium (DMEM), fetal bovine serum (FBS), trypsin-EDTA, L-glutamine, and antibiotics were purchased from Gibco BRL (Grand Island, NY, USA). The Aroclor 1254 induced male Sprague Dawley rat liver S9 fraction was obtained from Molecular Toxicology Inc. (Boone, NC, USA). All other chemicals were of analytical grade and were obtained from standard commercial suppliers.

### 2.2. Sample and Analysis by GC-MS

*O. majorana* dried leaves, acquired from Luar Sul Company (Santa Cruz do Sul, RS, Brazil) with an Egyptian Certificate of Origin and free of macroscopic contaminants, were used. The essential oil was obtained by steam distillation using a modified Clevenger device. The GC-MS was carried out on a Shimadzu mass spectrometer (GC/MS-QP5000) connected with cylindrical quadruple and operated at 70 eV ionization energy. An apolar Durabond-DB5 column (30 m × 0.25 mm × 0.25 *μ*m) was used. To confirm the identity of the compounds, a polar column was also used (LM-120). The temperature was programmed from 60 to 300°C at 3°C/min and the injector and detector temperatures were set at 220°C and 250°C, respectively. GC-FID was used for quantification [16, 17]. The relative composition of the oils was obtained by electronic integration and the identification of the compounds was based on comparison to retention indices, determined relatively to the retention times of an homologous series of n-alkanes, and mass spectra of commercial database (NIST) and literature [18].

### 2.3. Bacterial and Mammalian Strains

*Salmonella typhimurium *strains TA98, TA97a, TA1535, TA100, and TA102 were obtained from Molecular Toxicology Inc. (Boone, NC, USA). TA98 detects frameshifts in the DNA target –C–G–C–G–C–G–C–G; TA97a detects frameshift mutations in –C–C–C–C–C–C–, +1 cytosine; TA1535 detects base pair substitutions and its corresponding isogenic strain TA100 detects base pair substitutions of a leucine [GAG] by proline [GGG]; and TA102 strain is sensitive to oxidative and alkylating mutagens and detects transversions or transitions in TAA DNA sequences [19].

Chinese hamster lung fibroblast V79 cells were obtained from Banco de Células do Rio de Janeiro (Rio de Janeiro, Brazil). V79 cells were chosen in this study because this cell line is largely adopted in cytogenetic assays mainly because of its stable karyotype and relatively short cellular cycle, varying between 12 and 16 h [20].

### 2.4. *Salmonella*/Microsome Mutagenicity Assay

Mutagenicity was assayed by the preincubation procedure where cells were incubated with and without metabolic activation in five strains of* S. typhimurium*. The S9 metabolic activation mixture (S9 mix) was prepared according to Maron and Ames [19]. Briefly, 100 *μ*L of test bacterial cultures (1-2 × 10^9^ cells/mL) was incubated at 37°C with different concentrations of* O. majorana* essential oil in the absence or in the presence of S9 mix (4% S9 fraction, 5 mM D-glucose-6-phosphate, 4 mM NADP, 33 mM MgCl_2_/8 mM KCl, and 100 mM phosphate buffer pH 7.4, as concentrations per tube) for 20–30 min, without shaking. Subsequently, 2 mL of soft agar (0.6% agar, 0.5% NaCl, 50 *μ*M histidine, and 50 *μ*M biotin, pH 7.4, 42°C) was added to the test tube and poured immediately onto a plate of minimal agar (1.5% agar, Vogel-Bonner E medium, containing 2% glucose).

Aflatoxin B_1_ (1 *μ*g/plate) was used as positive control in the presence of metabolic activation (with S9 mix) for TA97a, TA98, and TA100 strains and 2-aminofluorene (2-AF, 10 *μ*g/plate) was used for TA102 and TA1535 strains. In the absence of metabolic activation, 4-nitroquinoline-oxide (4-NQO, 0.5 *μ*g/plate) was used for TA97a, TA98, and TA102 strains and sodium azide (1 *μ*g/plate) was used for TA100 and TA1535 strains. Plates were incubated in the dark at 37°C for 48 h before counting the revertant colonies.

### 2.5. *In Vitro* Mammalian Cell Micronucleus Test (MNvit)

V79 cells were cultivated in standard conditions with high glucose DMEM medium supplemented with heat-inactivated 10% FBS and antibiotics. Cells were maintained in 25 cm^2^ culture bottles at 37°C and humid atmosphere with 5% CO_2_. For harvesting and culture establishment, PBS (phosphate buffer saline, pH 7.4) and 0.25% trypsin-EDTA were used. For the experiments,* O. majorana* essential oil was diluted in DMSO and further diluted in DMEM culture medium obtaining different concentrations in which 5 *μ*L/mL was used as the highest test concentration. This concentration was chosen considering the extract solubility in the culture medium and the OECD suggestion for mutagenesis analysis [21]. The final concentration of DMSO in the cultures was 0.5%.

Thus, to verify the potential mutagenic effects of* O. majorana* essential oil, the MNvit assay was performed as described by Gonçalves et al. [22] and OECD 487 [21] with minor modifications. For this, 1 × 10^5^ V79 cells were seeded per well in 6-well plates and exposed to 0.003125, 0.00625, 0.0125, and 0.025 *μ*L/mL of* O. majorana* essential oil for 3 or 21 h. 0.5% DMSO was used as negative control and 0.75 *μ*g/mL colchicine and 0.250 *μ*g/mL etoposide were used as positive controls. In the 3 h treatment experiments, the medium was removed after treatment and replaced with fresh medium containing 3 *μ*g/mL cytochalasin B to block the cytokinesis and then incubated for 21 h (period corresponding to 1.5–2 normal cell cycles). In the 21 h of treatment experiments, cultures were exposed to* O. majorana* essential oil in the presence of 3 *μ*g/mL cytochalasin B. In both experiments, after the cytochalasin incubation, the cells were incubated in hypotonic solution (KCl 0.075 M) for 3 min at 4°C, prefixed and fixed with methanol and acetic acid (3 : 1) solution. This process was repeated and the cells were stored at 4°C overnight. Fixed cells were dropped on to microscope slides and stained with 2% Giemsa. The slide analysis was performed by means of a semiautomated scoring PathFinder Screen Tox system (IMSTAR, France) [23] or manually scored in optical microscope. It is important to note that the semiautomated scoring was previously validated in the laboratory demonstrating high correlation with the manual scoring results. Thus, for the analysis, the cell viability was first evaluated by the replication index [21]. For this, the number of mononucleated, binucleated, and multinucleated cells was counted in 500 cells/slide (manual analysis) or in all cells localized in a preselected area by slide (semiautomated analysis). For the analysis of micronucleus formation, at least 500 binucleated cells were considered for the presence of micronucleus, totalizing around 3000 cells per test group (two slides per treatment well, three wells per treatment group) when manual scoring was used. In the semiautomated scoring, the system identified in a preselected area the number of binucleated cells and the number of binucleated cells with micronucleus. Then, the technician confirmed the real presence of micronuclei in each binucleated cell previously identified by the software.

### 2.6. Statistical Analysis

The results of the* Salmonella*/microsome mutagenicity assay were analyzed by using the* Salmonella *Assay Software (Environmental Monitoring System Laboratory, EPA, software version 2.3). This program allows assessment of the dose-response effect by calculating the analysis of variance (ANOVA) between the measurements of the average number of revertants tested at different concentrations and the average number of revertants per plate from the negative control (mutagenic index, MI). A test substance was considered mutagenic when significant dose response and ANOVA variance were observed, and the increase in the mean number of revertants on test plates was at least twofold higher than that observed in the negative control plates (or MI ≥ 3 for TA1535 strain).

In the MNvit test, all experiments were independently repeated at least three times, with duplicate samples for each treatment. The results are expressed as mean ± standard deviation (SD) and were analyzed by one-way ANOVA followed by Dunnett's Multiple Comparison Test or Student's* t*-test when needed by using GraphPad Prism 5.0 software (GraphPad Inc., San Diego, CA). *p* < 0.05 was considered statistically significant.

## 3. Results and Discussion

### 3.1. Essential Oil Characterization

The analysis of the chemical composition of samples obtained from different geographical locations indicates that the biological activity is directly related to the concentration of the essential oil components, which may vary according to the region [4, 7, 24, 25]. Moreover, season, climate, stage of plant development at harvest, and the technique of extraction of the product may influence the quantity of the plant compounds [24, 26].

Fifteen compounds were identified in the* O. majorana* essential oil (Table 1). The most abundant compounds were *γ*-terpinene (25.73%), *α*-terpinene (17.35%), terpinen-4-ol (17.24%), and sabinene (10.8%). This chemical profile is in accordance with what is reported in the literature, with some quantitative variations. Rodrigues et al. [27] and Vági et al. [13] also reported the presence of terpenes as the major components of the* O. majorana* essential oil. Usually, terpinen-4-ol and *γ*-terpinene are described as the most abundant compounds in* O. majorana* essential oil and sabinene and *α*-terpinene are also observed [4, 7, 13, 24, 25].

### 3.2. Evaluation of Mutagenesis in Bacteria

The dose range of the* O. majorana* essential oil was determined in range finding experiments using the TA100 strain, with or without S9 mix, using five serially diluted concentrations (0.008–5 *μ*L/plate), where the highest dose tested was determined by the solubility of the substance and as suggested by the Ames Test Guideline (Table 2) [28]. Cytotoxicity (mutagenic index ≤0.6) can be observed in Table 3 at the highest concentrations used for TA98, TA100, TA102, and TA1535 in absence of metabolic activation and for TA100 in presence of metabolic activation. In regard to the range finding study, it was performed only in TA100 strain in accordance with OECD suggestion.

Thus, in the mutagenicity test the dose ranged between 0.0025 and 0.04 *μ*L/plate in the absence of S9 mix and between 0.005 and 0.08 *μ*L/plate in the presence of S9 mix. It can be seen in Table 3 that no mutagenic effect was detected on strains TA98 and TA97a in the presence or absence of metabolic activation. In addition, no mutagenicity was seen in the strain TA1535 and its corresponding isogenic strain TA100. Negative results were also observed in the TA102 strain, which is sensitive to oxidative and alkylating mutagens [19].

### 3.3. Evaluation of Mutagenesis in Mammalian Cells

The MNvit assay is a genotoxicity test for the detection of micronuclei in the cytoplasm of interphase cells [29] and was carried out to evaluate the potential of the* O. majorana *essential oil to induce chromosome mutation. The* O. majorana *essential oil concentrations were chosen based on replication index previously analyzed at 0.0005, 0.005, 0.05, 0.5, and 5 *μ*L/mL (Table 4) with 3 h of exposure, where a concentration-dependent decrease in cellular viability was observed mainly in concentrations ≥0.05 *μ*L/mL. Moreover, 100% cytotoxicity was observed at the concentrations of 0.5 and 5 *μ*L/mL. The analysis of micronucleus formation was performed in concentrations lower than 0.05 *μ*L/mL to provide the appropriate range of cytotoxicity (up to 55 ± 5%).

**Figure 1:**
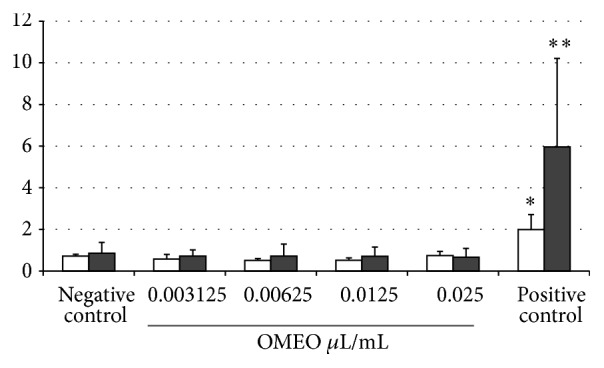
Micronucleus frequency of V79 cells exposed to different* O. majorana *essential oil (OMEO) concentrations for 3 h (white columns) and 21 h (black columns). Negative control: DMSO (0.5%); positive control: 0.75 *μ*g/mL colchicine (3 h) and 0.25 *μ*g/mL etoposide (21 h). ^*∗*^ *p* < 0.05; ^*∗∗*^ *p* < 0.01, in relation to the negative control (Student's* t*-test).

Micronuclei may originate from acentric chromosome fragments (i.e., lacking a centromere) or whole chromosomes that are unable to migrate to the poles during the anaphase stage of cell division. The assay detects the activity of clastogenic and aneugenic chemicals in cells that have undergone cell division during or after exposure to the test substance ([29]; reviewed in [21]). As illustrated in Table 5, the* O. majorana *essential oil did not decrease the cell viability after 3 h or 21 h of exposure. In relation to micronucleus formation in binucleated cells,* O. majorana *essential oil was not able to induce increase in the micronucleus frequency when compared to the negative control, in both periods evaluated (Figure 1). It is important to note that these results were observed in the absence of S9-mix and additional studies in the presence of metabolic activation are needed.

Several studies have reported that sweet marjoram leaves, especially in the form of an herbal tea, may be useful as immunostimulant and in reducing genotoxicity in patients under chemotherapeutic interventions, besides inhibiting the production of free radicals [3, 6]. Indeed, Khan et al. [12] proposed that reducing the availability of the genotoxic metabolites of chemical carcinogens from the body, by forming more water soluble conjugates, will consequently decrease the chance of an interaction with DNA through adduct formation that may lead to cancer.

Terpinen-4-ol, one of the main compounds found in* O. majorana* essential oil, is also the main active compound of the* Melaleuca alternifolia* essential oil. Both the* M. alternifolia* essential oil and the terpinen-4-ol compound were able to induce caspase-dependent apoptosis of melanoma cells [30]. Gomes-Carneiro et al. [31] reported that *α*-terpinene was not able to induce mutagenicity in* Salmonella* strains when tested alone.

In conclusion, the findings from the present study indicated that, at the concentrations and conditions used,* O. majorana* essential oil is not able to induce* in vitro* gene (*S. typhimurium *strains) and chromosome (V79 Chinese hamster lung fibroblast cells) mutations, respectively, contributing to the knowledge in the safe use of this essential oil. Further studies are now in progress to explore possible reproductive toxicity, teratology, and development toxicology induced by* O. majorana* essential oil* in vivo*.

## Figures and Tables

**Table 1:** Chemical composition of the *Origanum majorana* essential oil.

Compound	RI^a^	%
*α*-Thujene	921	3.96
*α*-Pinene	927	1.24
Sabinene	966	10.80
Myrcene	986	2.08
*α*-Phellandrene	1000	1.70
*α*-Terpinene	1012	17.35
*o*-Cymene	1019	2.24
*β*-Phellandrene	1023	7.05
*γ*-Terpinene	1053	25.73
Terpinolene	1082	3.76
N.I.^b^	1097	1.06
Terpinen-4-ol	1174	17.24
*Trans*-sabinene hydrate acetate	1248	0.13
Linalool acetate	1251	1.38
Terpinenen-4-ol acetate	1293	0.88
*(Z)*-caryophyllene	1404	2.72
N.I.^b^	1480	0.67
Total identified		98.26

^a^Relative retention index experimentally determined against n-alkanes on Durabond-DB5 column. ^b^Compound not identified.

**Table 2:** Range finding study of the *O. majorana* essential oil using the TA100 strain.

Substance	Concentration	Without metabolic activation (−S9)		With metabolic activation (+S9)	
		Rev/plate^a^	MI^b^	Rev/plate^a^	MI^b^
NC^a^	—	141.33 ± 17.56	—	148.00 ± 4.58	—
Essential oil	0.008 *µ*L/mL	105.67 ± 22.01	0.75	145.33 ± 18.15	0.98
	0.04 *µ*L/mL	53.00 ± 9.64	0.38^b^	127.00 ± 19.47	0.86
	0.2 *µ*L/mL	33.00 ± 23.07	0.23^b^	72.67 ± 13.05	0.49^b^
	1 *µ*L/mL	0.33 ± 0.58	0.00	0.33 ± 0.58	0.00
	5 *µ*L/mL	0.00 ± 0.00	0.00	0.00 ± 0.00	0.00

^a^Negative control: DMSO (10 *µ*L). ^b^Cytotoxicity.

**Table 3:** Induction of *his*+ revertants in *S. typhimurium *strains by *Origanum majorana* essential oil with and without metabolic activation (S9 mix).

Substance	Concentration	*S. typhimurium *strains									
		TA98		TA97a		TA100		TA1535		TA102	
		Rev/plate^a^	MI^b^	Rev/plate^a^	MI^b^	Rev/plate^a^	MI^b^	Rev/plate^a^	MI^b^	Rev/plate^a^	MI^b^
Without metabolic activation (−S9)											
NC^c^	—	19.7 ± 8.2	—	69.7 ± 3.1	—	103.7 ± 6.1	—	4.3 ± 2.5	—	291.7 ± 10.5	—
Essential oil (*µ*L/plate)	0.0025	24.7 ± 6.4	1.03	91.7 ± 7.5	1.32	102.3 ± 9.3	0.99	4.7 ± 2.1	1.09	268 ± 12.5	0.92
	0.005	14.5 ± 3.4	0.74	84.7 ± 18.9	1.22	110.7 ± 10.5	1.07	6.3 ± 4	1.45	255.3 ± 26.5	0.88
	0.01	15.2 ± 6.2	0.77	89.3 ± 23	1.28	95.7 ± 15.6	0.92	2 ± 0	0.46	253 ± 46.5	0.87
	0.02	10.5 ± 2.3	0.53	71.7 ± 15.5	1.03	75.5 ± 30.4	0.73	4 ± 1.7	0.92	174.7 ± 15.6	0.60
	0.04	9.2 ± 2.5	0.47	72 ± 13.1	1.03	41.7 ± 4	0.40	1.3 ± 1.2	0.31	111.3 ± 9.1	0.38
PC^d^ (*µ*g/plate)	0.5 (4NQO)	332 ± 22.6^*∗∗∗*^	20.68	464.3 ± 91.8^*∗∗∗*^	6.66	—	—	—	—	1031 ± 93.5^*∗∗∗*^	3.54
	1 (NaN_3_)	—	—	—	—	427.3 ± 31.2^*∗∗∗*^	4.12	312.3 ± 9^*∗∗∗*^	72.13	—	—

With metabolic activation (+S9)											
NC^c^	—	15.3 ± 1.5	—	100.7 ± 9.9	—	179.7 ± 11.1	—	9.3 ± 2.9	—	325.7 ± 15.5	—
Essential oil (*µ*L/plate)	0.005	19 ± 3.6	1.24	109.3 ± 8.5	1.09	155.7 ± 10	0.87	10.3 ± 4.5	1.1	310 ± 3.6	0.95
	0.01	23.3 ± 1.5	1.52	112 ± 20.1	1.11	141.3 ± 12.6	0.79	8 ± 3	0.86	338 ± 12.7	1.04
	0.02	15.7 ± 1.5	1.01	115 ± 10.5	1.14	135.3 ± 6.7	0.75	7.7 ± 1.5	0.83	332.7 ± 29	1.02
	0.04	21 ± 9.5	1.37	113.7 ± 8.7	1.13	112 ± 14.9	0.62	8.3 ± 3.5	0.89	301.7 ± 26	0.93
	0.08	18.7 ± 1.2	1.22	131.7 ± 10.6	1.31	99.3 ± 14	0.55	7.7 ± 2.3	0.83	295.7 ± 31.1	0.91
PC^e^ (*µ*g/plate)	1 (AFB_1_)	1587 ± 91.1^*∗∗∗*^	103.5	587 ± 88.5^*∗∗*^	5.83	660.7 ± 59.9^*∗∗*^	3.68	—	—	—	—
	10 (2-AF)	—	—	—	—	—	—	104.3 ± 12.1^*∗∗*^	11.18	767.3 ± 53.6^*∗∗∗*^	2.36

^a^Number of revertants/plate: mean of three independent experiments ± SD; ^b^MI: mutagenic index (number of *his*+ induced in the sample/number of spontaneous *his*+ in the negative control). ^c^Negative control: DMSO (10 *µ*L) used as solvent for the extract. ^d^Positive control (−S9): NaN_3_ (sodium azide) to TA100 and TA1535 and 4-NQO to TA97a, TA98, and TA102. ^e^Positive control (+S9): AFB_1_ (aflatoxin B_1_) for TA97a, TA98, and TA100 and 2-aminofluorene for TA102 and TA1535; ^*∗∗*^ *p* < 0.01; ^*∗∗∗*^ *p* < 0.001, in relation to the negative control.

**Table 4:** Replication index of cultures exposed for 3 h to *Origanum majorana* essential oil.

Substance	Concentration	Replication factor (mean ± S.D.)	Replication index (%)
NC^a^	—	0.809 ± 0.012	100
Essential oil	0.0005 *μ*L/mL	0.823 ± 0.027	102
	0.005 *μ*L/mL	0.800 ± 0.035	99
	0.05 *μ*L/mL	0.126 ± 0.028	15
	0.5 *μ*L/mL	0 ± 0	0
	5 *μ*L/mL	0 ± 0	0

^a^Negative control: DMSO (0.5%).

**Table 5:** Replication index of cultures exposed to *Origanum majorana* essential oil for 3 h or 21 h.

Substance	Concentration	3 h		21 h	
		Replication factor (mean ± S.D.)	Replication index (%)	Replication factor (mean ± S.D.)	Replication index (%)
NC^a^	—	0.726 ± 0.021	100	0.820 ± 0.050	100
Essential oil	0.003125 *μ*L/mL	0.735 ± 0.019	101	0.822 ± 0.036	100
	0.00625 *μ*L/mL	0.698 ± 0.021	96	0.809 ± 0.014	99
	0.0125 *μ*L/mL	0.731 ± 0.012	101	0.837 ± 0.027	102
	0.025 *μ*L/mL	0.722 ± 0.031	99	0.769 ± 0.024	94
Colchicine^b^	0.75 *μ*g/mL	0.501 ± 0.013	69	—	—
Etoposide^b^	0.25 *μ*g/mL	—	—	0.767 ± 0.028	94

^a^Negative control: DMSO (0.5%). ^b^Positive control.
